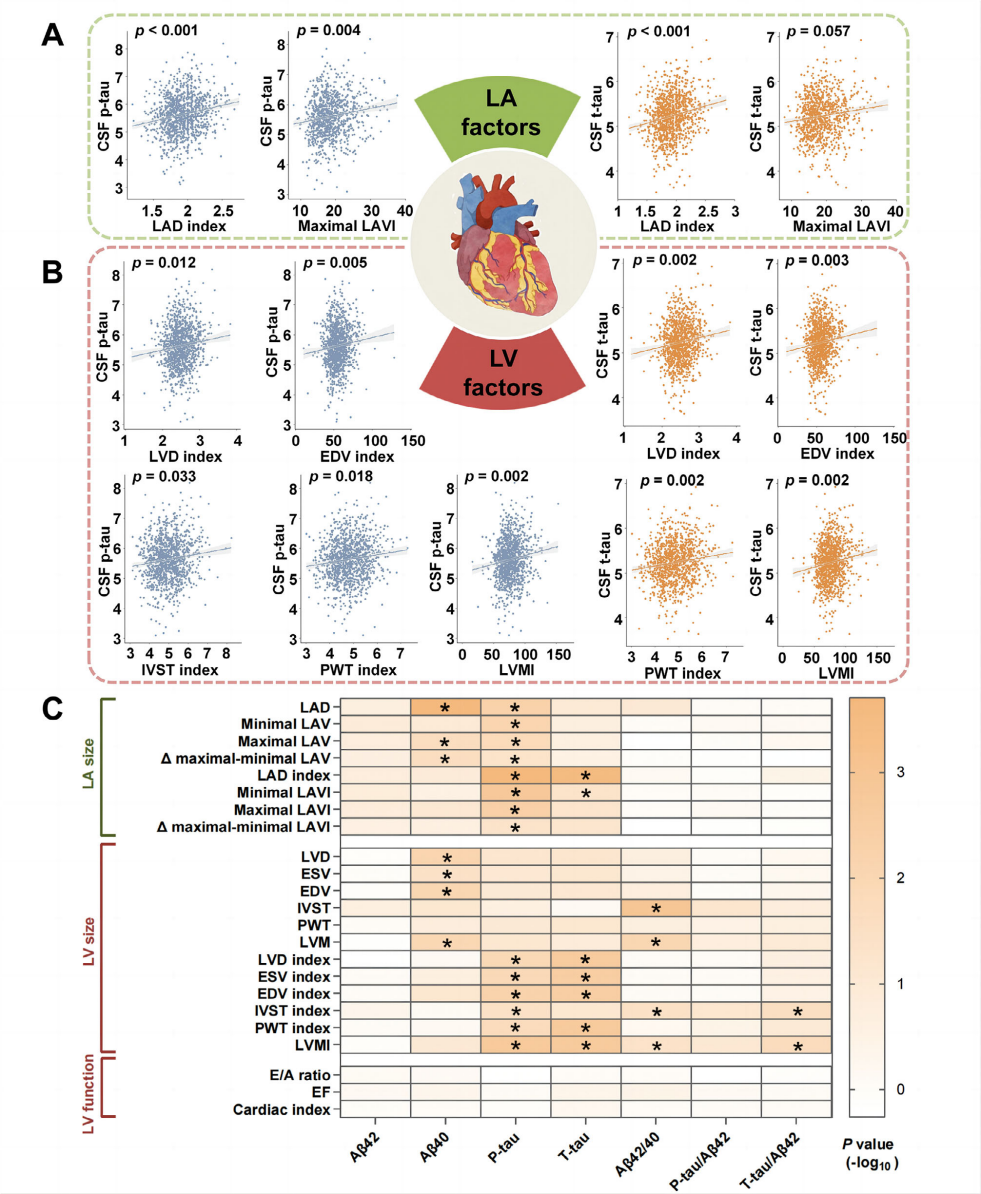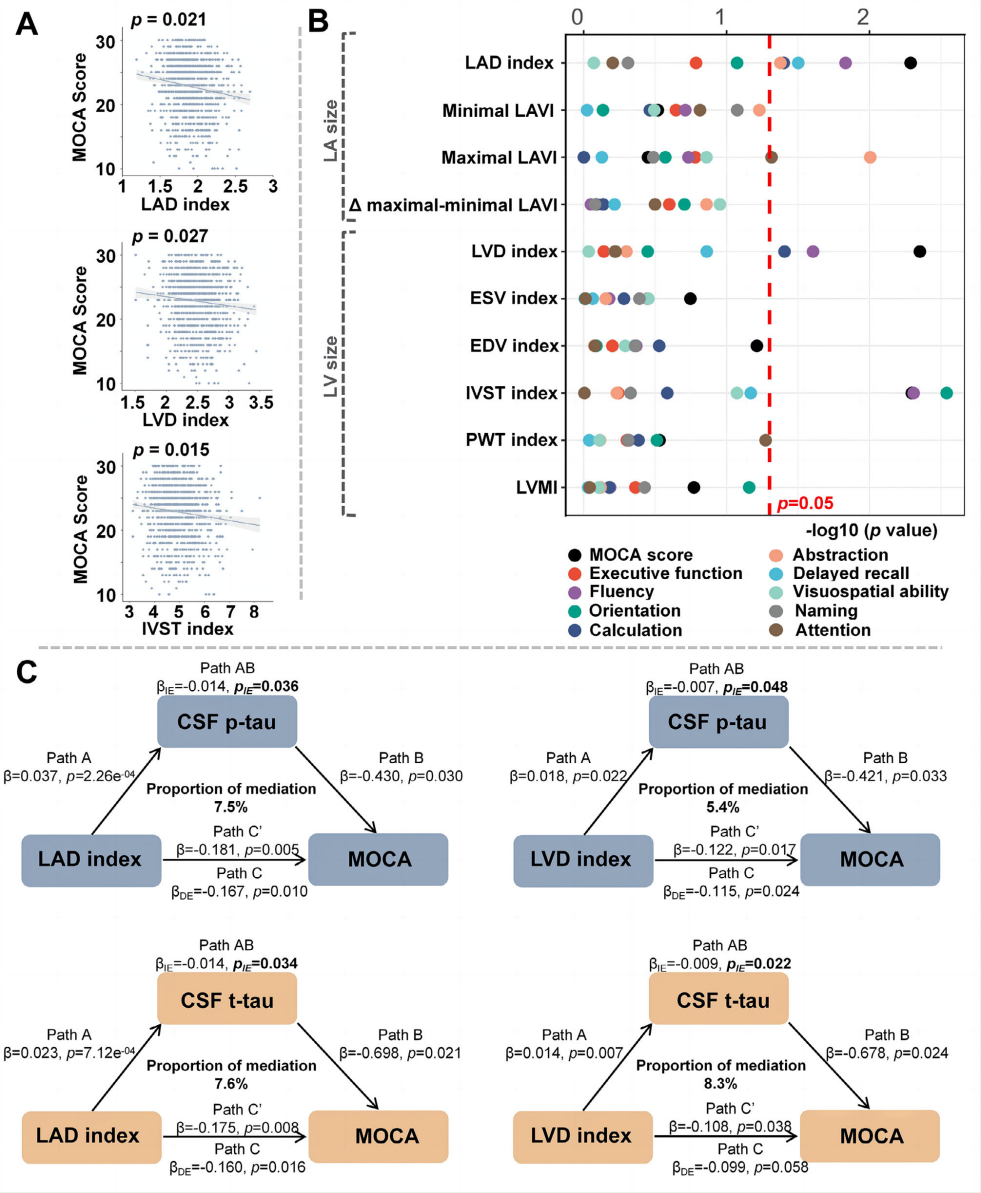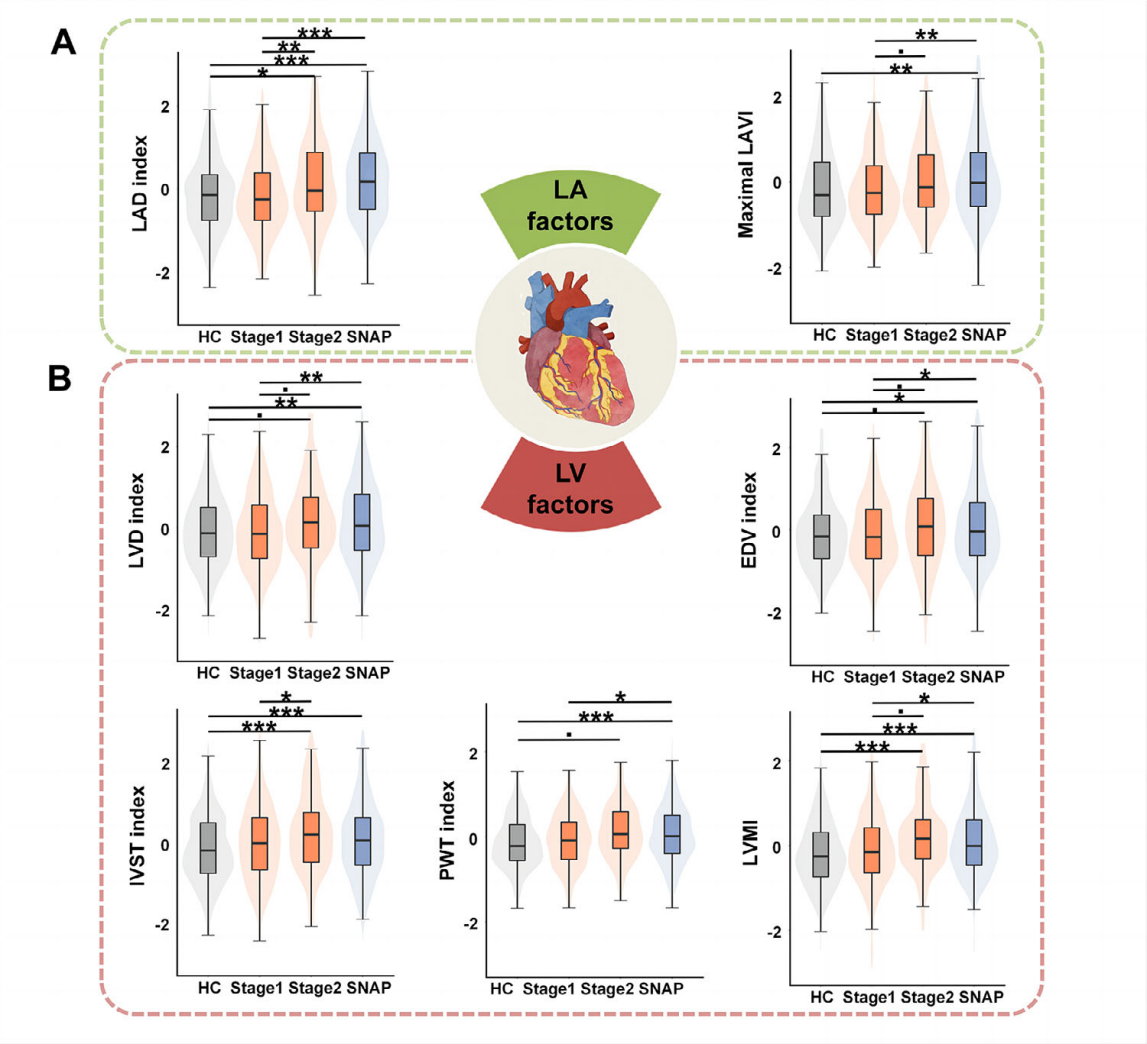# Echocardiographic measures of the left heart and cerebrospinal fluid biomarkers of Alzheimer's disease pathology in cognitively intact adults: The CABLE study

**DOI:** 10.1002/alz.086077

**Published:** 2025-01-09

**Authors:** He‐Ying Hu, Hao Hu, Jin‐Tai Yu

**Affiliations:** ^1^ Qingdao University, Qingdao China; ^2^ Qingdao Municipal Hospital, Qingdao University, qingdao, Shandong China; ^3^ National Center for Neurological Disorders, Shanghai, Shanghai China; ^4^ Huashan Hospital, Fudan University, Shanghai, Shanghai China

## Abstract

**Background:**

The heart‐brain connection has been proposed to correlate cardiac disorders with brain health. However, the associations between subclinical alterations in cardiac structure or function and Alzheimer's disease (AD) pathologies haven't been fully elucidated. This study aimed to delineate the interrelationships between the subclinical alterations in the left heart, cerebrospinal fluid (CSF) AD biomarkers, and cognition.

**Method:**

Multiple linear regression analyses were conducted in 1,244 cognitively normal participants (mean age=65.5 years; 43% female) who had measurements of the left heart on echocardiograms (left atrial [LA] and left ventricular [LV] morphologic or functional parameters) and CSF AD biomarkers. Covariates included sociodemographic variables, lifestyle or other cardiovascular characteristics, and apolipoprotein E4 status. The mediating effects of AD pathology on heart‐cognition relationships were examined. Besides, differences in the cardiac parameters across the ATN categories were tested using ANOVA and logistic regression models.

**Result:**

LA and LV enlargement (captured by increased diameters and volumes) and LV hypertrophy (captured by increased interventricular septal or posterior wall thickness and ventricular mass) were significantly associated with higher CSF levels of phosphorylated (p)‐tau and total (t)‐tau, as well as poorer cognitive function. Tau pathologies mediated the heart‐cognition relationships. Besides, left heart parameters were higher in stage 2 and suspected non‐Alzheimer's pathology groups than in healthy controls. Moreover, the accumulation of cardiac alterations further magnified their damage to the brain.

**Conclusion:**

These findings suggested the close associations of subclinical cardiac changes with tau hyperphosphorylation, neurodegeneration and cognitive dysfunction. This study provided a comprehensive insight into the left heart condition and its impact on AD pathologies. The echocardiographic measurements of the left heart might help to identify the patients at risk of AD continuum, and we should early monitor the cardiac alterations in older adults to prevent dementia or AD.